# Improving Patient Flow in the Surgical Assessment Unit by Facilitating Access to Computer Tomography Scanning

**DOI:** 10.7759/cureus.30010

**Published:** 2022-10-06

**Authors:** Amjad Burgan, Ysabelle Embury-Young

**Affiliations:** 1 Trauma and Orthopaedics, Yeovil District Hospital National Health Service (NHS) Foundation Trust, Yeovil, GBR; 2 Ear, Nose, and Throat, University Hospitals Bristol National Health Service (NHS) Foundation Trust, Bristol, GBR

**Keywords:** length of admission, access to imaging, computer tomography scanning, surgical assessment unit, patient flow

## Abstract

Introduction

Patient flow is “the ability of healthcare systems to manage patients effectively and with minimal delays as they move through stages of care”. National Health Service (NHS) England has highlighted the importance of improving patient flow, and the many benefits to both staff and patients, including: “improved clinical outcome and experience of patients”; “eliminating waits and delays”; “saving time and effort by avoiding duplication of work”; “saving money”; and “improving the reputation of the NHS”. Limited access to computerised tomography (CT) imaging for surgical patients can disrupt patient flow through delayed diagnosis, management, and discharge. The aims of this quality improvement project were to assess the effect of a dedicated Surgical Assessment Unit (SAU) radiologist on access to CT scanning and its impact on patient flow. The measured outcomes were time from admission to CT scanning, and length of admission.

Method

In June 2020, a radiology registrar was allocated to the SAU to vet and report CT imaging. CT requests from the SAU were identified from Picture Archiving and Communication System (PACS) in the first two weeks of June 2019 and 2020 during normal working hours (Monday to Friday, between 0900 and 1700). We performed a retrospective audit and compared data from 2019 and 2020. The measured primary outcome was time from admission to imaging. The secondary outcome was the length of admission.

Results

In the two-week period in June 2019, 25 patients requiring a CT scan presented to SAU during normal working hours. In 2020, this number increased to 40 patients. In 2019, 5 patients had a CT scan performed within four hours of admission (20%). In 2020, following the allocation of a radiologist to the SAU, 33 patients (82.5%) had their CT performed within four hours of admission. In 2019, one patient (4%) was discharged within six hours of admission. A further 16% had a length of admission between six and 12 hours. The majority of patients (80%) remained in the hospital for more than 12 hours. In 2020, 45% of patients were discharged within four hours following a CT scan. More than half of the patients (57.5%) were discharged within 12 hours.

Conclusion

The allocation of a radiologist in the SAU led to both a reduction in time to perform CT scans and an increased number of patients being discharged within 12 hours. These results suggest that early access to imaging improves patient flow. Further research will help to establish whether the allocation of a radiologist in the SAU is a beneficial and cost-effective method of improving patient flow, and whether this may help reduce the current pressure on NHS services.

## Introduction

Patient flow is “the ability of healthcare systems to manage patients effectively and with minimal delays as they move through stages of care” [1]. In the context of current pressures on primary and secondary care services, focus on patient flow is paramount to ensure safe and timely patient care [2,3]. National Health Service (NHS) England has highlighted the importance of improving patient flow, and the many benefits to both staff and patients, including: “improved clinical outcome and experience of patients”, “eliminating waits and delays”, “saving time and effort by avoiding duplication of work”, “saving money” and “improving the reputation of the NHS" [4]. As an acute service, the surgical assessment unit (SAU) “should have ready access to diagnostics to support patient care and early discharge” in order to facilitate patient flow [1]. Lack of access to prompt CT imaging and reporting can lead to a delay in the diagnosis, management, and discharge of a patient, acting as a bottleneck in the patient pathway and leading to an increased length of admission [5,6]. There is also a huge variation in imaging reporting times between hospitals putting patients at risk of harm [7]. Published quality improvement projects have demonstrated that the provision of an SAU-specific Ultrasound Scanning service can lead to a reduction in the length of admission [8,9]. There is also evidence that early CT scanning of patients with abdominal pain can lead to a reduction in the length of admission [10].

The aim of this project was to determine the effect of a dedicated radiology registrar on access to CT and patient flow. The measured outcomes are the time from admission to CT scanning and the length of admission.

## Materials and methods

This research was carried out at the Royal Gwent Hospital in Newport. This District General Hospital, with more than 3,400 staff and 700 beds, serves a population of 600,000 [11]. In October 2019, a dedicated radiology registrar was placed in the SAU to facilitate access to imaging by vetting and reporting CT scans. This service was available from the hours of 0900 to 1700.

All CT requests from the Surgical Assessment Unit were identified from Picture Archiving and Communication System (PACS) in two weeks of June 2019 (10/06/2019 to 23/06/2019) and June 2020 (08/06/2020 to 21/06/2020). Information collected included: imaging type, patient hospital identification number, date and time of admission, imaging report, and discharge. Patients admitted between 0900 to 1700 Monday to Friday were included in the analysis. Using this information we were able to calculate the time from admission to the CT scan and the total length of admission. Due to a small data sample, a further power analysis was not performed. Results are presented in absolute numbers of patients and percentage of patients in a given time period. Patients with incomplete data were excluded. No ethical approval was required.

## Results

In the two-week period in June 2019, 25 patients requiring a CT scan presented to SAU during normal working hours. In 2020, this number increased to 40 patients. The first measured outcome was the time from admission to the CT scan. In 2019, five patients had a CT scan performed within four hours of admission. This accounts for 20% of all patients who had CT imaging during normal working hours. The majority of patients (44%) had their CT scan performed between four and 8 hours after admission. In 2020, following the allocation of a radiologist to the SAU, 33 patients (82.5%) had their CT performed within four hours of admission. Within eight hours of admission, 95% of patients requiring a CT scan had their imaging completed (Table 1).

**Table 1:** Time from Admission to CT scan (2019 and 2022) presented in absolute numbers and percentage of all CT scans CT scans performed in 2 weeks of June 2019/20 (Mon- Fri, 0900-1700)

	Number of CT scans Performed (n)		Percentage of CT scans performed (%)	
Time from admission to CT scan (hrs:mins)	2019	2020	2019	2020
00:00 to 04:00	5	33	20	82.5
04:00 to 08:00	11	5	44	12.5
08:00 to 12:00	0	0	0	0
≥ 12:00	9	2	36	5

The second measured outcome was the total length of admission for patients requiring CT imaging. In 2019 only one patient was discharged within six hours of admission. A further four patients had a length of admission between six and 12 hours. The majority of patients (80%) remained in the hospital for more than 12 hours. In 2020, 45% of patients were discharged within four hours following a CT scan. More than half of the patients (57.5%) were discharged within 12 hours (Table 2).

**Table 2:** Length of admission (2019 and 2022) presented in absolute numbers and percentage of total patients

	Number of patients discharged (n)		Percentage of patients discharged (%)	
Length of admission (hrs:mins)	2019	2020	2019	2020
00:00 to 06:00	1	18	4	45
06:00 to 12:00	4	5	16	12.5
≥ 12:00	20	17	80	42.5

## Discussion

These results demonstrate an improvement in both measured outcomes, with reduced time from admission to imaging and reduced length of admission. Overall, the number of CT scans performed increased from 25 to 40 between 2019 and 2020. The possible reasons for this are discussed below and include the closure of another local hospital and the decision-making of different radiologists. Interestingly, the percentage of CT scans performed within four hours of admission also increased (20.0% to 82.5%). Although demand for CT imaging increased, there was enough provision to ensure that the increased demand was met in a timely manner. This suggests that having an allocated radiology registrar within the SAU does improve the provision of CT scans for patients, reducing the time between admission and imaging.

The number of patients who had a CT scan and were discharged within six hours also increased (4% to 45%). It is possible that the additional provision of a radiology registrar on SAU in 2020 allowed earlier imaging to take place and therefore earlier decision-making from the surgical team in the patient pathway. This would lead to earlier discharge decisions being made and subsequently improved patient flow. In the context of rising pressures on secondary care services, these results suggest that the allocation of radiologists in the SAU may be an important tool in improving patient flow and bed capacity.

Limitations

There are a number of limitations identified in this study. The overall number of cases from 2019 and 2020 is small, and therefore any conclusions may be of limited power. The number of CT scans in the two-week period increased between 2019 and 2020. There are confounding factors that may have contributed to this. For example, the radiologist vetting scan requests in 2019 is likely to have differed from the radiologist in 2020. Dependent on the radiologist placed in SAU they may have differing opinions on which imaging modality is best and this may alter the number of CTs performed [12]. Additionally, the presenting complaint of patients within the two-week period is likely to have differed and it may be CT imaging was more relevant to those patients in 2020.

Another significant confounding factor was the merger of another local hospital SAU with Royal Gwent SAU in late 2019 [13]. This led to an increase in the number of patients presenting at the Royal Gwent and may account for the increase in the number of CT scans performed per week. Other possible sequelae are an increase in senior-led decision-making and increased pressure on bed space leading to reduced time in decision to discharge or admission to maintain SAU capacity [14].

Finally, the placement of a radiologist in SAU relies on adequate staffing and the availability of a room with access to imaging viewing and reporting software. This may not be feasible or replicable in other hospitals. Indeed the NHS in England has a backlog of over 600,000 patients awaiting imaging, and diverting staff to the SAU may cause further delays in other areas [15]. There is also the associated cost of staffing and setting up the service, however, this may be offset by the reduced length of admission.

## Conclusions

These results align with current literature and suggest that early access to imaging has a positive effect on patient flow. Our results demonstrate that the allocation of a radiologist in the SAU led to improved access to CT imaging in a timely manner, and therefore may help achieve the targets set out in the NHS Improvement Good Practice Guide. Further research is required to assess whether this improvement is replicable and applicable to other surgical admission units.

The current NHS crisis of capacity is an opportunity to implement change to improve efficiency for now and the future. These changes are often inspired by the innovation of local departments and hospitals. The allocation of a radiologist in the SAU may be one measure to help reduce the current pressure on NHS services and improve patient care
